# Assessment of Virgin Olive Oil Adulteration by a Rapid Luminescent Method

**DOI:** 10.3390/foods8080287

**Published:** 2019-07-25

**Authors:** Raúl González-Domínguez, Ana Sayago, María Teresa Morales, Ángeles Fernández-Recamales

**Affiliations:** 1 Department of Chemistry, Faculty of Experimental Sciences, University of Huelva, 21007 Huelva, Spain; 2 International Campus of Excellence CeiA3, University of Huelva, 21007 Huelva, Spain; 3 Department of Analytical Chemistry, Faculty of Pharmacy, University of Seville, 41012 Seville, Spain

**Keywords:** virgin olive oil, hazelnut oil, adulteration, luminescence

## Abstract

The adulteration of virgin olive oil with hazelnut oil is a common fraud in the food industry, which makes mandatory the development of accurate methods to guarantee the authenticity and traceability of virgin olive oil. In this work, we demonstrate the potential of a rapid luminescent method to characterize edible oils and to detect adulterations among them. A regression model based on five luminescent frequencies related to minor oil components was designed and validated, providing excellent performance for the detection of virgin olive oil adulteration.

## 1. Introduction

Industrial processing of vegetable oils usually includes a refining step that removes almost all of the undesirable minor components, such as colored compounds, free fatty acids, or metals, but retaining the major neutral lipids and most of the natural antioxidants [1]. However, virgin olive oil is consumed crude, thus conserving the flavor compounds, vitamins, and other important natural components. Virgin olive oil adulteration is an important issue since the sensory and nutritional properties of this gourmet oil are responsible for its expensive price [2]. The most common adulteration process is the mixing of virgin olive oil with other cheaper oils, such as refined olive oil, seed oils (e.g., sunflower, soybean, corn, canola), as well as walnut, peanut, and hazelnut oils. This fraud is not easily detectable since refined oils lack of many of the minor compounds usually employed to authenticate olive oil, and the sensory characteristics of virgin oils cannot be significantly altered by this blending [3]. Particularly, hazelnut oil is commonly used for olive oil adulteration since its detection in mixtures is very challenging due to the very similar chemical profiles of these two oils [4]. This adulteration has been estimated to cause a loss of four million Euros per year for countries in the European Union. Therefore, there is a great interest in the development of accurate analytical methods, in combination with powerful chemometric tools [5], to guarantee the authenticity and traceability of virgin olive oil, and to detected olive oil adulteration. Frauds are usually detected by applying chromatographic techniques based on the determination of trans-fatty acids, sterols, triglycerides, hydrocarbons, and other components [6,7,8]. Other authors have also described the application of thermal analysis [9], and electroanalytical techniques [10,11,12,13], as potential approaches for investigating olive oil authenticity. Alternatively, spectroscopic techniques have also been proposed as suitable alternatives to traditional methodologies given their inherent advantages, including rapidity, environment-friendly nature, and low sample size requirement [14,15,16]. Several authors have described the application of vibrational spectroscopy (near-infrared spectroscopy, NIR; Fourier transform mid-infrared spectroscopy, FT-MIR; Fourier transform Raman spectroscopy, FT-Raman), NMR and visible absorption spectroscopy to investigate oil adulteration [17,18,19]. Among them, fluorescence spectroscopy enables rapid spectral acquisition with lower signal-to-noise ratio, so the combination of this technique with advanced chemometric tools has been successfully applied for the detection of olive oil adulteration in previous studies [20,21]. In this vein, the aim of this work was to evaluate the possibilities of a rapid luminescent method to detect fraudulent mixtures of virgin olive oils with refined hazelnut oils.

## 2. Materials and Methods

### 2.1. Oil Samples

Forty samples of four types of edible vegetable oils were directly acquired from oil mill stores from various locations: Eighteen virgin olive oil samples (VOO) of different European varieties (cvs. Cornicabra, Picual, Hojiblanca, Arbequina and Verdial were obtained from different producers in Spain; Cima di Bitonto and Tzunnati were of Italian and Greek origin, respectively; and a commercial sample (Borges brand, a blend of several olive oil varieties Tàrrega, Spain) were acquired in a market from Huelva in 2015), eight refined olive oils (ROO), seven virgin hazelnut oils (VHZO), and seven refined hazelnut oils (RHZO) (hazelnut oils were obtained from Turkish producers). Picual and Cima di Bitonto mixtures with refined hazelnut oils were prepared in the concentration range 5–30% (5, 10, 15, 20, 25 and 30% w/w) to evaluate the response of the luminescent spectra to the addition of adulterants. A test set was also prepared in the same concentration range using Cornicabra olive oil and refined hazelnut oil. Binary mixtures were prepared in triplicate. Oil samples were kept at 4 °C and analyzed without any pre-treatment to avoid potential interferences.

### 2.2. Instrumentation

All measurements were performed using a RF-1501 Shimadzu spectrofluorophotometer (Shimadzu Corporation, Kyoto, Japan) equipped with a continuous 150 W xenon lamp, and excitation and emission monochromators. Fluorescence emission spectra (300–800 nm, 1 nm interval) were collected at 650 nm excitation wavelength, while excitation and emission slits were set at 10 nm. Samples were scanned using a 3 mL non-fluorescent cell (10 mm path-length). After each series of measurements, the cuvette was cleaned using detergent, followed by a rinse with deionized hot water and acetone in order to dry and eliminate any remaining fat. Each sample was analyzed in triplicate. The spectrofluorophotometer was interfaced to a computer for spectral acquisition and data processing.

### 2.3. Data Analysis

Univariate and multivariate statistical analyses were conducted in Statistica 8.0 (Stat Soft, Tulsa, Oklahoma) and SIMCA-P™ software (version 11.5, UMetrics AB, Umeå, Sweden). Pattern recognition analysis was carried out by using unsupervised (principal component analysis, PCA; multidimensional scaling, MDS) and supervised (linear discriminant analysis, LDA; soft independent modeling of class analogy, SIMCA) chemometric techniques. One-way ANOVA was applied for the selection of wavelengths able to differentiate oil samples, and the response to the addition of adulterants was evaluated by stepwise linear regression analysis (SLRA). Before performing statistical analysis, data was submitted to different pretreatments and combination of pretreatments. Standard normal variate (SNV) transformation and first derivative were selected as the most suitable scaling procedures to remove undesirable factors in the spectral raw data and to correct for possible baseline shifts in the spectral data. First derivative was performed according to the Savitzky and Golay method with second-order smoothing polynomials through five points.

## 3. Results and Discussion

### 3.1. Spectral Characterization of Oil Samples

**Figure 1:**
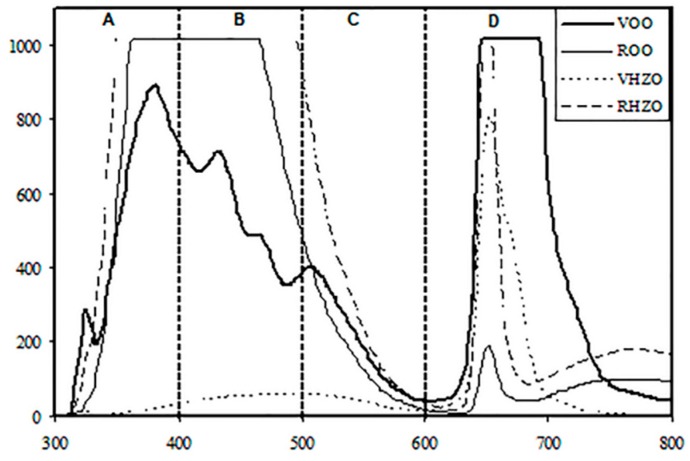
Characteristic luminescent spectra of virgin olive oil, virgin hazelnut oil, refined olive oil and refined hazelnut oil.

Figure 1 shows the characteristic luminescent spectra of extra virgin olive, refined olive, crude hazelnut, and refined hazelnut oil samples, which can be divided in four regions. Region A (300–400 nm) is mainly related to tocopherols and pigments and, as can be seen in Figure 1, allowed distinguishing virgin olive oils from the other edible oils studied in this work. Region B (400–500 nm) showed good correlation with conjugated dienes (K232), conjugated trienes (K270), and hydrolysis products [22], which are associated with oil quality. This might explain the clear discrimination between refined and non-refined oils in this luminescent region. Vitamin E is correlated with region C (500–600 nm) [22], thus making it possible to distinguish virgin olive oil from virgin hazelnut oil and refined oils. Finally, region D (600–800 nm) is mainly attributed to chlorophylls and pheophytines, pigments usually contained in virgin olive oil [23].

The method precision was estimated by computing the standard deviations (SD) and relative standard deviations (RSD) for each spectrum wavelength. However, results shown in Table 1 are presented as mean values for each of the regions from the spectrum. Repeatability was assessed by analyzing five replicates of a Cornicabra oil sample within the same day, while internal reproducibility was estimated by acquiring three replicate spectra of two different virgin olive oil samples in five different days by two different analysts. As shown in Table 1, repeatability and internal reproducibility were excellent for all the regions studied, with RSD values lower than 2% and 15%, respectively.

### 3.2. Classification of Oils

**Figure 2:**
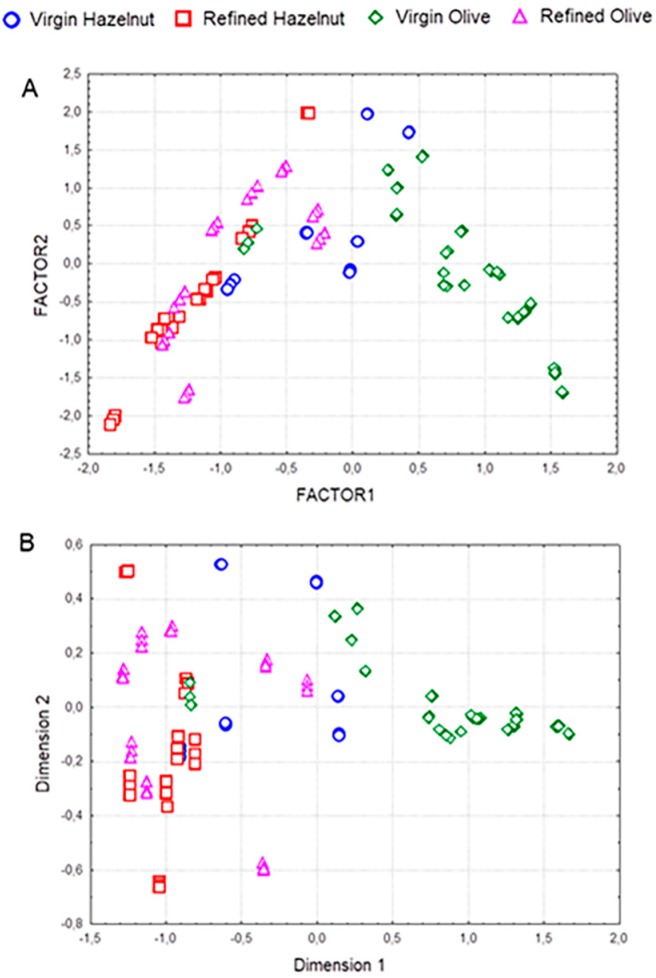
Principal component analysis (PCA) score plot (**A**) and multidimensional scaling plot (**B**) showing the distribution of samples from the four study groups in the plane defined by the two first principal components.

As a first exploratory step, principal component analysis (PCA) and multidimensional scaling (MDS) were applied for a preliminary evaluation of data quality. PCA is an unsupervised method for reducing the dimensionality of the original data matrix retaining the maximum amount of variability, thus enabling us to get an overview of the data to identify possible outliers and trends towards the grouping of samples. On the other hand, the goal of MDS is to detect meaningful underlying dimensions that allow explaining observed similarities or dissimilarities between the investigated objects [24]. First, the acquired spectra were processed by using several spectral pretreatments, including first derivative, standard normal variate (SNV), and both first derivative and SNV. PCA was then performed on processed and normalized data, considering only factors with eigenvalues higher than 1 (Kaiser criterion). The best goodness of fit and validity was obtained by using spectral data in the region 650–800 nm (R^2^_X_ = 0.999 and Q^2^_cumulative_ = 0.998). This PCA model explained 98% of the total variance with four principal components (PC1 59.9%, PC2 28.3%, PC3 8.1%, and PC4 1.9), with PC1 being mainly associated with wavelengths in the ranges 656–720 and 752–800 nm. Figure 2A shows the distribution of samples in the plane defined by the first two principal components, which explained 88.2% of the original variance. Thus, it can be observed that samples were clustered in two groups, the first one comprising virgin olive oil samples, located in the right side of the plot, while the second cluster showed a high degree of overlapping between the other three groups of samples. The application of MDS also provided a clear separation of virgin olive oils from the rest of the sample set (Figure 2B). Dimension one discriminated between virgin olive oil samples and all refined samples as well as virgin hazelnut ones. Refined hazelnut and refined olive oils were clustered together, while virgin hazelnut samples were distributed in two groups, the first one near to the refined group, comprising three roasted samples, and the second one closer to the virgin olive oil cluster, constituted by three non-roasted crude hazelnut oil samples.

**Figure 3:**
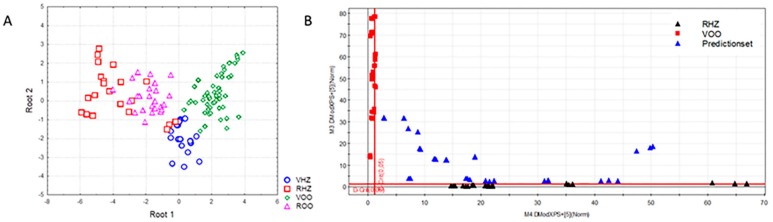
Linear discriminant analysis (LDA) score plot (**A**) and Coomans plot (**B**).

To achieve a more reliable differentiation among oil classes, various supervised pattern recognition procedures were also applied to the data matrix, including linear discriminant analysis (LDA) and soft independent modeling of class analogy (SIMCA). These classification methods have been successfully employed in previous studies to discriminate extra virgin olive oils according to variety and geographical origin [25,26], thus presenting a great potential in assessing food adulteration. LDA is a supervised classification tool based on the generation of orthogonal linear discriminant functions equal to the number of categories minus one. In this work, stepwise LDA was applied to classify oils according to the four categories, and the most significant variables involved in sample differentiation were selected using a Wilks’ λ and F value as criterion for inclusion or removal of variables in the model. The best results were obtained when LDA was carried out on normalized data in the range 752–800 nm, with a mean prediction ability of 93%. The model retained seven variables (F to enter = 3.00 and F to remove = 1.00), showing a clear distinction among virgin oil samples and refined oil samples (Figure 3A). On the other hand, SIMCA is a class modeling technique that builds class models based on significant principal components of category, and classifies samples on the basis of their distance from the model representing each category. Models with five PCs were obtained by using SNV-pretreated data mean (99.7% of predictive ability), which explained 99.5, 99.9, 99.7, and 100% of the variance for virgin hazelnut, refined hazelnut, virgin olive, and refined olive categories, respectively. As shown in the corresponding Coomans plots, all vegetable oil samples could be correctly assigned (Figure 3B).

### 3.3. Detection of Virgin Olive Oil Adulteration by Regression Analysis

The mixture of virgin olive oil with refined hazelnut oil is a common fraud since this adulteration is not easily detectable by tasting or smelling. For this reason, we conducted an ANOVA test to look for variables enabling the differentiation between these two oils. Only wavelengths attributable to pigments (i.e., chlorophyll, pheophytin) and tocopherols presented significant *p*-values, in line with previous results [20].

**Figure 4:**
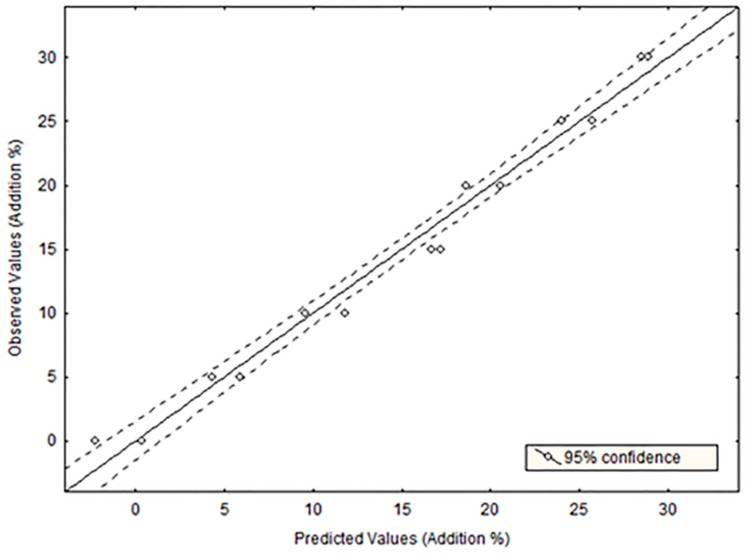
Prediction of adulteration percentage applying stepwise multiple linear regression to virgin olive oil and refined hazelnut oil mixtures.

Subsequently, regression models were built to assess the potential of luminescence to detect olive oil adulteration, for which several binary mixtures were prepared and analyzed. To this end, stepwise multiple regression analysis (SLRA) was applied to mixtures prepared by adding refined hazelnut oil in the concentration range 5–30%. This studied concentration range was selected on the basis of previous reports demonstrating the difficulty of detecting this adulteration at low concentration levels [4]. Two sets of blends were prepared by using Picual and Cima di Bitonto virgin olive oils, which were selected on the basis of ANOVA results to cover maximum (Picual) and minimum (Cima di Bitonto) values for pigments and tocopherols. SLRA was applied to these sets of mixtures under the strictest conditions (F to enter = 8.00 and tolerance = 0.01) to select the variables to be included in the model. Thus, five wavelengths were selected (319, 446, 476, 685, and 704 nm) to get an adjusted-R^2^ of 0.972 (R^2^ = 0.98; *p* = 0.000001; Durbin-Watson d statistic = 2.02). The linearity of the model (Figure 4) was excellent in the studied concentration range (5–30% w/w), with slope and intercept values close to 1 and 0, respectively. The statistical validity of this model was assessed by ANOVA through the lack of fit F tests. The ratio between the mean square due to the lack of fit and the mean square due to the pure experimental error was calculated (F = 2.45) and compared with the tabulated F value (F = 2.93), thus evidencing good adjustment between the observed and predicted values. Thus, the root mean square error of calibration (RMSEC) computed for this SLRA model was 0.77.

Finally, a test sample was also prepared by mixing Cornicabra virgin olive oil with refined hazelnut oil in the same concentration range in order to evaluate the predictive ability of the model. The validation of the regression equation (Figure 4) in these test samples yielded adjusted-R^2^ = 0.99 (R^2^ = 0.99; *p* = 0.00079). Then, the root mean square error of prediction (RMSEP) was calculated as a measure of the accuracy of the model to predict the response. In the present work, the SLRA model yielded the RMSEP = 1.15, thus demonstrating the excellent accuracy of the luminescent method to detect adulterations. The limit of detection (LOD), calculated as three times the standard deviation of the intercept divided by the validation curve slope, was equal to 2.3%.

### 3.4. Method Performance: Comparison with Traditional Approaches

To evaluate the suitability and advantages of the luminescent method here presented to detect virgin olive oil adulteration with hazelnut oil, Table 2 shows the performance of previously published methods in this field. Due to the chemical similarities between olive and hazelnut oils chromatographic and mass spectrometric methods focused on the determination of specific oil components, such as filbertone [27], Maillard products [28], phytosterols [29], tocopherols [30], fatty acids [31], proteins [32], and phospholipids [33], usually provide low sensitivity to detect this adulteration. Furthermore, these methods are usually time consuming and require the application of complex extraction procedures, thus hindering their implementation in the food industry practice. Alternatively, several authors have also proposed the use of spectroscopic approaches, including nuclear magnetic resonance, infrared, and Raman spectroscopy [34,35,36,37], with increased potential for detecting relatively low adulteration levels (5–10%, w/w). Similar performance has been described for some genetic methods based on the application of polymerase chain reaction and subsequent capillary electrophoresis analysis (PCR-CE) [31], or high resolution melting (PCR-HRM) [38].

The luminescent method developed in this work yielded limits of detection around 2%, clearly surpassing the performance of most of the previously described methodologies. Furthermore, it should be also noted the greater simplicity, low analytical cost, and rapidity of analysis of this luminescent method compared with other conventional approaches, thus facilitating its implementation in routine laboratory analysis. Therefore, the present study demonstrates the possibilities of luminescent methods for the genuineness assessment of virgin olive oil.

## 4. Conclusions

In this work, we have developed a rapid and simple luminescent method, in combination with advanced chemometric tools, to characterize and classify edible vegetable oils with good prediction ability. Furthermore, a regression model based on five luminescent frequencies related was validated for sensitive detection of virgin olive oil adulteration with hazelnut oil, a common fraud in food industry. The main strengths of this methodology rely on the simplicity, fast and low cost analysis compared with conventional approaches for adulteration detection. As a limitation, it should be mentioned the non-automatable nature of this technique, which could be improved in the future by the use of flow-through fluorescence cuvettes. Therefore, this work clearly demonstrates the possibilities of luminescent methods for the genuineness assessment of virgin olive oil, potentially implementable in food industry and regulatory agencies as a routine tool for adulteration detection.

## Figures and Tables

**Table 1:** Method repeatability (r) and internal reproducibility (R).

		Spectral Regions			
		300–400 nm	400–500 nm	500–600 nm	600–800 nm
**r**	**SD**	3.72	1.84	2.86	2.77
	**RSD (%)**	1.3	0.43	1.81	1.21
**R**	**SD**	14.96	22.27	17.33	6.07
	**RSD (%)**	10.69	6.01	7.63	3.34

**Table 2:** Performance of previously published methods to detect virgin olive oil adulteration with hazelnut oil.

Method	Lowest Level of Adulteration Detected (w/w)	Reference
Chromatographic methods		
LC/GC (filbertone)	20–25%	[27]
LC (Maillard products)	5%	[28]
GC (phytosterols)	>30%	[29]
LC (tocopherols)	3%	[30]
GC (fatty acids)	Non detectable	[31]
Mass spectrometric methods		
MALDI-TOF-MS (proteins)	20%	[32]
MALDI-TOF-MS (phospholipids)	1%	[33]
Spectroscopic methods		
^1^H/^31^P NMR	5%	[34]
2D NMR	6.27%	[35]
FTIR	25%	[36]
FT-Raman, FT-MIR	8%	[37]
Genetic methods		
PCR-CE	5%	[31]
PCR-HRM	10%	[38]
